# Resveratrol, a polyphenol phytoalexin, protects against doxorubicin-induced cardiotoxicity

**DOI:** 10.1111/jcmm.12633

**Published:** 2015-07-14

**Authors:** Jun Gu, Wei Hu, Da-dong Zhang

**Affiliations:** a Department of Cardiology, Shanghai Ninth People’s Hospital, Shanghai Jiaotong University School of MedicineShanghai, China; b Department of Cardiology, Shanghai Minhang Hospital, Fudan UniversityShanghai, China

**Keywords:** doxorubicin, cardiotoxicity, resveratrol, ROS, apoptosis, autophagy

## Abstract

Doxorubicin is the mainstay of treatment for various haematological malignancies and solid tumours. However, its clinical application may be hampered by dose-dependent cardiotoxicity. The mechanism of doxorubicin-induced cardiotoxicity may involve various signalling pathways including free radical generation, peroxynitrite formation, calcium overloading, mitochondrial dysfunction and alteration in apoptosis and autophagy. Interestingly, the use of resveratrol in combination with doxorubicin has been reported to prevent cardiac toxicity as well as to exert a synergistic effect against tumour cells both *in vivo* and *in vitro*. Thus, the aim of this review is to summarize current knowledge and to elucidate the protective effect of resveratrol in doxorubicin-induced cardiotoxicity.

- Introduction
- Resveratrol alleviates cardiomyocytes oxidative stress induced by doxorubicin
- Resveratrol mitigates cardiomyocytes apoptosis induced by doxorubicin
- Resveratrol modulates doxorubicin-induced cardiocyomytes autophagy
- Resveratrol ameliorates doxorubicin-induced cardiac fibrosis
- Effects of resveratrol on the anti-tumour capacity of doxorubicin
- Summary

## Introduction

Doxorubicin is an effective anthracycline antibiotic used to treat a broad range of haematogenous and solid malignancies, but its clinical use is limited by its dose-dependent side effects, namely irreversible degenerative cardiomyopathy and congestive heart failure 1. Doxorubicin induced-cardiotoxicity may occur immediately after a single dose or several weeks to months after repetitive doxorubicin administration. The mechanism of doxorubicin-induced cardiotoxicity is complex and may involve various signalling pathways including free radical generation, peroxynitrite formation, calcium overloading, mitochondrial dysfunction, apoptosis and autophagy 1.

Epidemiological studies have demonstrated that the incidence of cardiovascular disorders in France is strikingly lower as compared with other western countries with a fat-containing diet. This so-called ‘French paradox’ has been attributed to moderate consumption of red wine in France. Resveratrol (trans-3,5,4′-trihydroxystilbene), a polyphenol compound found in grapes and red wine in significant amounts, has been designated as the responsible agent of the ‘French paradox’. In animals, resveratrol has been shown to have numerous beneficial effects, such as promoting vasodilation in models of coronary heart disease and enhancing the expression of antioxidant enzymes 2. In addition, resveratrol can protect the heart against ischaemia-reperfusion injury 3, improve endothelial function 4 and prevent platelet aggregation 5. Interestingly, the use of resveratrol in combination with doxorubicin has been reported to prevent doxorubicin-induced cardiotoxicity as well as to exert a synergistic effect against tumour cells both *in vivo* and *in vitro* 6–12. It is noteworthy that the cardioprotective effect of resveratrol is associated with reduced oxidative stress, inhibited apoptosis and modulated autophagy process 6–12. This article discusses the protective effect of resveratrol in doxorubicin-induced cardiotoxicity.

## Resveratrol alleviates cardiomyocytes oxidative stress induced by doxorubicin

Available laboratory evidence suggests that increased oxidative stress with increased free radical production and decreased myocardial endogenous antioxidants plays an important role in the pathogenesis of doxorubicin-induced cardiotoxicity 9,13. Increases in lipid peroxidation and myeloperoxidase activity as a result of the toxic effects of doxorubicin accompanied by significant reductions in glutathione level, antioxidant capacity and Na,K-ATPase activity were found in the cardiac tissue 9. Resveratrol treatment was shown to prevent the severity of doxorubicin-induced cardiotoxicity by alleviating the extent of oxidative stress 9, as demonstrated by increased levels of superoxide dismutase (SOD) and decreased levels of malondialdehyde, suggesting its free radical scavenging capacity 14. It was reported that the increase in reactive oxygen species (ROS) production stimulated by doxorubicin was localized to the mitochondria in cardiomyocytes and resveratrol pre-treatment prevented doxorubicin-induced mitochondrial damage 7.

Multiple mechanisms are involved in resveratrol antioxidant activities. First, resveratrol improves mitochondrial function through reduced basal ROS generation, increased MnSOD and SIRT1 (sirtuin 1) activity, and subsequent polarization of mitochondrial membrane potential 7,15. Second, it has been shown that resveratrol can inhibit nicotinamide adenine dinucleotide phosphate (NADPH)- and adenosine 5′-diphosphate-Fe^+^-lipid peroxidation. Third, resveratrol markedly up-regulates transcription of nuclear factor-E2-related factor-2 target gene NADPH: quinone oxidoreductase 1, gamma-glutamylcysteine synthetase, and haeme oxygenase-1 (HO-1), which can attenuate cellular oxidative stress 16.

## Resveratrol mitigates cardiomyocytes apoptosis induced by doxorubicin

Increased myocardial cell apoptosis is another proposed mechanism by which doxorubicin induces cardiotoxicity 17. Mitochondrial dysfunction is one of the most critical events associated with doxorubicin-induced cardiomyocyte apoptosis, in which anti-apoptotic Bcl-2 and pro-apoptotic Bax genes are suggested to play a major role in determining cell’s survival or death after apoptotic stimuli. Previous study has indicated that the p53 tumour suppressor gene may use transcriptional activation to regulate the gene products of the Bcl-2 family proteins, which then promotes caspase-3–mediated apoptosis 8. Caspase-independent apoptosis pathway is also involved in doxorubicin-induced cardiomyocytes apoptosis, which is associated with the mitochondrial release of apoptosis-inducing factor, a flavoprotein with NADH oxidase activity, located in the mitochondrial inter-membrane space 18.

Accumulated lines of evidence suggest that resveratrol acts as an anti-apoptotic agent, providing cardioprotection through inhibition of caspase-3 expression and activity 14,19. Our previous study showed that doxorubicin injection attenuated HO-1 expression and activity as well as increased p53 expression, modulated Bcl-2/Bax expression and enhanced caspase-3 activity in lymphoma nude mice. These cardiotoxic effects of doxorubicin were ameliorated by its combination with resveratrol. However, the protective effects of resveratrol were reversed by the addition of HO-1 inhibitor (ZnPP). Taken together, it is concluded that HO-1 plays a core role for protective action of resveratrol in doxorubicin-induced cardiomyocytes apoptosis 12. Furthermore, it has been reported that resveratrol ameliorates doxorubicin-induced apoptosis in cardiomyocytes through the restoration of SIRT1 activity, which decreases the level of acetylated p53 and p53-dependent transcription of Bax 8,11.

## Resveratrol modulates doxorubicin-induced cardiocyomytes autophagy

Autophagy predominantly functions as a pro-survival pathway during nutrient deprivation and other forms of cellular stress. However, when autophagy is rigorously activated, the autophagic machinery might also be used for self-destruction, which might result in enhanced oxidative stress, decreased ATP production, collapse of the cellular catabolic machinery and hence, necrosis or apoptosis. Accordingly, autophagy in the myocardium has been viewed as a double-edged sword that can be maladaptive in one context and beneficial in another depending on the type and duration of the injury as well as the levels of autophagic activity 20.

The role of autophagy in doxorubicin-induced cardiotoxicity *in vitro* studies is still conflicting 10,21–26. Of those studies that demonstrated doxorubicin-induced up-regulation of autophagy, several reported that inhibition of autophagy by 3-methyladenine (3-MA), or other means of autophagy inhibition, improved cell viability 10,21–24. Of the studies that reported doxorubicin-induced suppression of autophagy, stimulation of autophagy by rapamycin or glucose-depletion led to enhanced cell viability 25,26. Moreover, the discrepancies between these studies do not appear to be doxorubicin dose (0.1–20 μM) or time (6–48 hrs) dependent.

Compared with the inconsistent results in *in vitro* studies, *in vivo* experiments should be put a high value. In an animal model of acute form of doxorubicin cardiotoxicity (single injection), doxorubicin per se increased the LC3-II/LC3-I ratio and p62 in the left ventricle, and the increase in LC3-II/LC3-I and p62 levels was because of an impairment of autophagic flux 6. Another study also revealed that in acute doxorubicin cardiotoxicity, autophagic function was impaired, resulting in the accumulation of LC3-II and p62 25. However, in the chronic form of doxorubicin cardiotoxicity (several injections within 2–4 weeks), doxorubicin-induced up-regulation of autophagy, and 3-MA strongly down-regulated the expression of beclin 1 in doxorubicin-induced failing heart and inhibited the formation of autophagic vacuoles 21. It was further observed that the strong autophagy response by doxorubicin exposure was paralleled with apoptosis and size decrease in cardiomyocytes. 3-MA inhibited the doxorubicin-induced autophagy and attenuated cardiomyocyte apoptosis and size decrease 27. Therefore, we speculate that doxorubicin might ameliorate autophagy in acute doxorubicin cardiotoxicity *in vivo*, while enhance autophagy in the chronic form.

Resveratrol has been shown to increase autophagy 6,28–30 as well as inhibit autophagy 10,31 in some specific circumstances. When cardiomyocyte autophagy is triggered by doxorubicin, resveratrol attenuates cardiotoxicity by suppressing autophagy, which is probably mediated through its inhibitory effect on S6K1 10. When doxorubicin decreases cardiomyocytes autophagy, resveratrol restored the impaired autophagic function 6. Furthermore, resveratrol attenuates cardiomyocyte apoptosis and oxidative stress injury in diabetic mice, associated with the restoration of impaired autophagic flux through SIRT1/FOXO1(forkhead box O1)/Rab7 axis 28. Thus, resveratrol might recover the dysregulation of cardiomyocyte autophagy induced by doxorubicin.

## Resveratrol ameliorates doxorubicin-induced cardiac fibrosis

It was reported that doxorubicin not only up-regulated transforming growth factor-beta1 (TGF-β1) gene expression but also increased fibrosis marker content and induced massive collagen fibres deposition in LV tissues, but these fibrotic effects of doxorubicin were ameliorated by its combination with resveratrol 14. In another study, resveratrol also exerted anti-fibrotic effects against cardiovascular remodelling in deoxycorticosterone acetate-treated rats 32.

## Effects of resveratrol on the anti-tumour capacity of doxorubicin

Whether resveratrol affects the anti-tumour capacity of doxorubicin has also been evaluated in some studies. Our previous group showed that resveratrol supplement had no impact on the anti-tumour capacity of doxorubicin in lymphoma nude mice 12. Pre-treatment with resveratrol increased the cell antioxidant ability by improving the activity of SOD, prevented or limited intracellular damage and ameliorated the harmful effects of ROS in 3T3 normal cells 33. In addition, resveratrol had synergistic effects with doxorubicin against MCF-7 (Michigan Cancer Foundation-7) breast cancer cells 33. Another study indicated that treatment with a combination of resveratrol and doxorubicin would be a helpful strategy for increasing the efficacy of doxorubicin by promoting an intracellular accumulation of doxorubicin and decreasing multi-drug resistance in tumour cells 34 as well as protecting against its cardiotoxicity 35. Therefore, it raises the possibility that the combined use of doxorubicin with resveratrol may be a viable chemotherapeutic modality that can selectively destroy tumours while concurrently limiting cardiac damage.

## Summary

**Figure 1:**
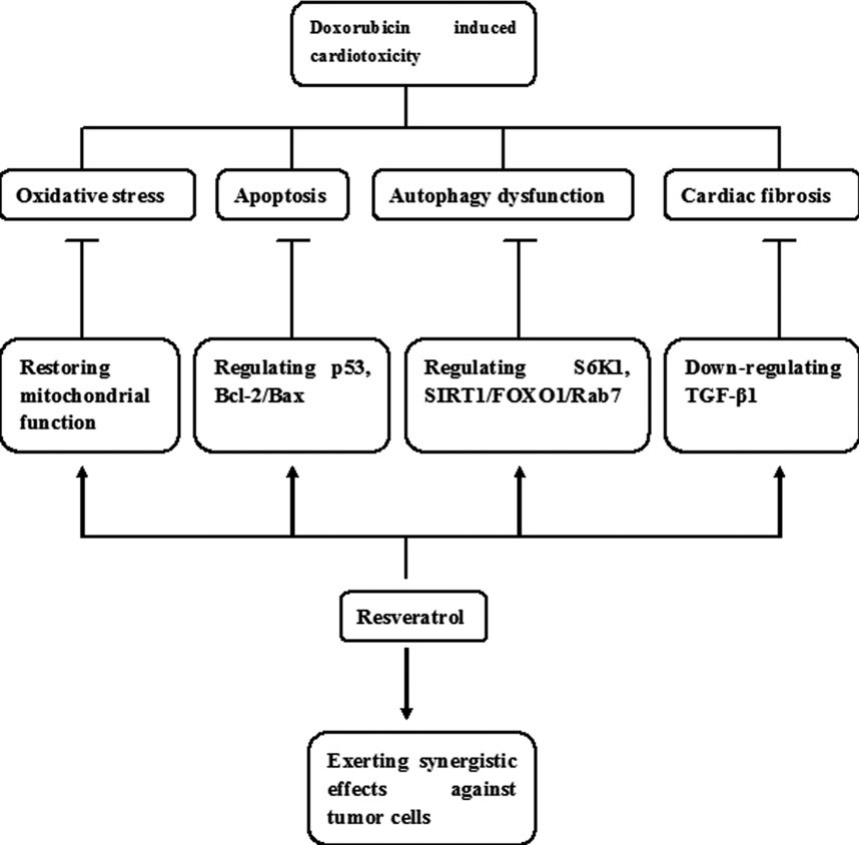
Proposed mechanisms of resveratrol protecting against doxorubicin-induced cardiotoxicity.

In conclusion, resveratrol exerts beneficial effect against doxorubicin-induced cardiotoxicity, which may involve various mechanisms including regulation of oxidant stress, apoptosis, autophagy and fibrosis. Resveratrol supplement has been shown to prevent doxorubicin-induced cardiac toxicity as well as to exert a synergistic effect against tumour cells (Fig. 1).
